# Mesenchymal Stem Cells Reversed Morphine Tolerance and Opioid-induced Hyperalgesia

**DOI:** 10.1038/srep32096

**Published:** 2016-08-24

**Authors:** Zhen Hua, LiPing Liu, Jun Shen, Katherine Cheng, Aijun Liu, Jing Yang, Lina Wang, Tingyu Qu, HongNa Yang, Yan Li, Haiyan Wu, John Narouze, Yan Yin, Jianguo Cheng

**Affiliations:** 1 Departments of Pain Management and Neurosciences, Lerner Research Institute and Anaesthesiology Institute, Cleveland Clinic, 9500 Euclid Avenue, Cleveland, Ohio 44195, USA; 2 Department of Anesthesiology, Beijing Hospital, No. 1 Dahua Road, Beijing 100730, China; 3 Psychiatric Institute, Department of Psychiatry, College of Medicine, University of Illinois at Chicago, Chicago, IL, USA

## Abstract

More than 240 million opioid prescriptions are dispensed annually to treat pain in the US. The use of opioids is commonly associated with opioid tolerance (OT) and opioid-induced hyperalgesia (OIH), which limit efficacy and compromise safety. The dearth of effective way to prevent or treat OT and OIH is a major medical challenge. We hypothesized that mesenchymal stem cells (MSCs) attenuate OT and OIH in rats and mice based on the understanding that MSCs possess remarkable anti-inflammatory properties and that both OT and chronic pain are associated with neuroinflammation in the spinal cord. We found that the development of OT and OIH was effectively prevented by either intravenous or intrathecal MSC transplantation (MSC-TP), which was performed before morphine treatment. Remarkably, established OT and OIH were significantly reversed by either intravenous or intrathecal MSCs when cells were transplanted after repeated morphine injections. The animals did not show any abnormality in vital organs or functions. Immunohistochemistry revealed that the treatments significantly reduced activation level of microglia and astrocytes in the spinal cord. We have thus demonstrated that MSC-TP promises to be a potentially safe and effective way to prevent and reverse two of the major problems of opioid therapy.

Chronic pain is a significant public health problem. It afflicts more than 100 million Americans and costs more than $635 billion annually^1^,^2^. Opioids, such as morphine, play an indispensable role in pain relief but are often associated with two major problems: opioid tolerance (OT) and opioid-induced hyperalgesia (OIH)^3^,^4^,^5^,^6^,^7^. OT is a physiological process where the body adjusts to a medication of frequent exposure and requires escalating doses to achieve the same effect. OIH is a phenomenon, in which individuals taking opioids to treat pain paradoxically develop an increased sensitivity to noxious stimuli. Both OT and OIH in animals have been validated in humans^8^,^9^. Nearly 50,000 people die every year of opioid overdose in the US, leading the Center for Disease Control and Prevention (CDC) to declare the problem an ongoing “national epidemic”. These facts underscore an urgent need for finding effective therapies to treat pain and OT and to reduce the disastrous outcomes associated with opioid treatment. Distinct molecular mechanisms are indicated for the two closely related but different phenomena^9^. Neuroinflammation, mediated by immune cells and glial cells, appears to play a central role^10^,^11^. Opioids such as morphine can cause neuroinflammation^12^ through acting on Toll-like receptor 4 on microglia and lead to development of OT^13^. Similarly, OIH is mediated by μ opioid receptor-dependent expression of P2X4 receptors on microglia and release of brain-derived neurotropic factor (BDNF)^14^. The P2X4-BDNF-TrkB pathway mediates microglia-to-neurons signaling and leads to sensitization of spinal lamina I neurons and OIH^14^. Thus, modulating neuroinflammation may prove to be an effective strategy to treat both OT and OIH.

We aim to develop a safe and efficacious therapy for OT and OIH in clinical practice. We chose to use MSCs because of their powerful paracrine functions, as shown in animal models of diseases such as traumatic brain injury^15^, peripheral neuropathy^16^,^17^, and neuropathic pain^18^. Immunomodulatory and anti-inflammatory effects of MSCs were related to neuroprotection, neuroregeneration, and neuroneuromodulation in these studies. For example, intravenous (IV) injection of human adipose-derived MSCs (hAD-MSCs) induced a significant reduction in mechanical allodynia and complete reversal of thermal hyperalgesia in a dose-dependent fashion in a mouse model of diabetic neuropathy^19^. The treatment decreased the level of IL-1β and increased IL-10 in the lesioned nerve and restored normal inducible nitric oxide synthase (iNOS) expression in the spinal cord. More recently, it was shown that IT rMSCs inhibited neuropathic pain via secretion of transforming growth factor beta (TGF-β)^20^. Thus, MSCs may release factors that promote tissue recovery through stimulating resident stem/progenitor cells, remodeling extracellular matrix, forming new blood vessels, and modulating immune functions^21^,^22^,^23^.

We hypothesized that MSC transplantation (MSC-TP) attenuates chronic OT that is induced by long-term daily morphine injections. We further hypothesized that MSC-TP attenuates OIH that is developed as a consequence of chronic morphine injections. We tested these hypotheses by using intrathecal and intravenous routes of transplantation in rats and mice and studied the distribution of the transplanted cells and the level of activation of microglia and astrocytes in the spinal cord in response to morphine and MSC-TP.

## Results

**Figure 1:**
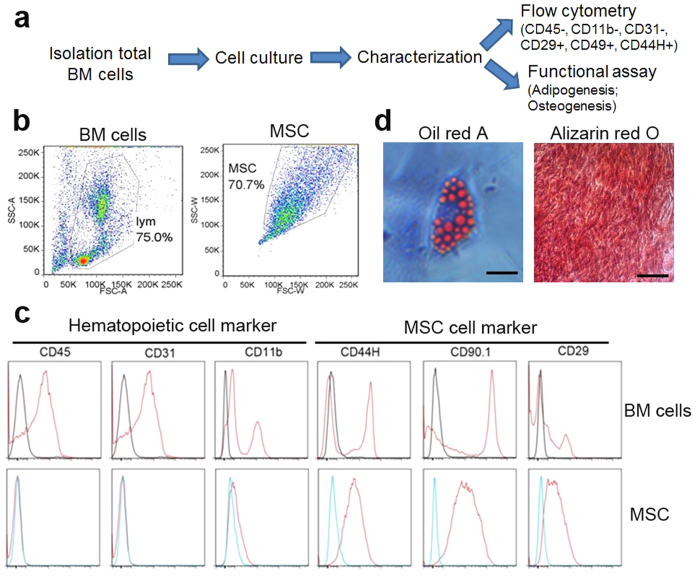
Isolation and characterization of MSCs from the rat bone marrow. (**a**) Experimental Scheme. (**b,c**) Flow cytometry data. (**b**) Different forward scatter (FCS) and side scatter (SSC) patterns between bone marrow (BM) cells (left), and bone marrow derived mesenchymal stem cells (MSCs) at passage 4 (right). **(c**) Cell surface markers (red) characteristic of hematopoietic cells and for MSCs respectively^68^. Unstained controls are indicated as blue. (**d**) MSCs were differentiated to adipose cells with lipid droplets accumulated in the cytoplasm stained with oil red (left) and osteoblast cells stained with Alizarin red (right) in respective media. These data represent three individual experiments.

We first isolated MSCs from rat bone marrow and characterized the cells through flow cytometry (FACS) and induced differentiations. These cells showed morphological properties and cell markers characteristic of stem cells and differentiated into osteoblast cells and adipose cells in specific culture media (Fig. 1).

We then tested the preventive and therapeutic effects of intrathecal and intravenous MSC-TPs on OT, which was induced by daily morphine injections. Acute OT was induced after 3 days of daily injections. Administration of cumulative doses of morphine on day 4 produced a dose-response curve with a maximum effect dose of 18 mg/kg (MS: 24.52 ± 0.48, n = 5), which was significantly higher than that of the control group (8.0 mg/kg) (NS: 25.00 ± 0, n = 3) (Supplementary Fig. 1a; P = 0.01). Chronic OT was induced by daily morphine injections for 3 to 4 weeks and evaluated by measuring two sets of paw withdrawal thresholds (PWTs) to mechanical and thermal stimulation. The first were measured before daily morphine injection and the second were measured 50 min after the injection. The differences between the two sets reflect the level of tolerance. A large difference indicates no or low tolerance while a small difference indicates high tolerance. The differences decreased gradually and significantly after 7 days of morphine treatment, reached a minimal difference at day 12, and maintained a small difference thereafter (Supplementary Fig. 1b, df = 7, F = 45.97; P < 0.0001; n = 8). OT was further evaluated by the tail flick test. Maximum possible effect (MPE) of morphine was used to indicate tolerance; high MPE (%) indicates low or no tolerance while low MPE indicates high tolerance. MPE was significantly reduced after 7 days of morphine treatment, reaching ~10% of the baseline value by day 12 (Supplementary Fig. 1c; df = 6, F = 47.34; P < 0.0001; n = 12).

**Figure 2:**
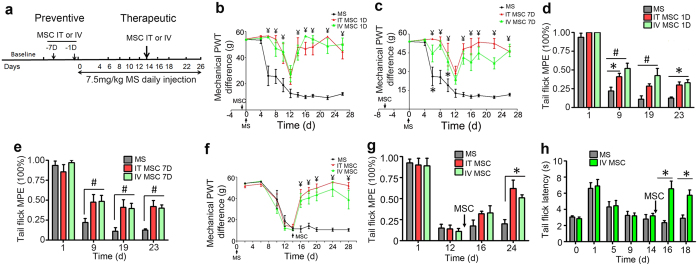
Preventive and Therapeutic effects of MSC transplantation (MSC-TP) on opioid tolerance (OT). **(a**) Experimental scheme. MSC-TP was performed either intravenously (IV) or intrathecally (IT) 1 day (**b,d**) or 7 days (**c,e**) before the initiation of daily MS injections. Pain-like behaviors in rats were assessed by von Frey filament (**b,c)** and tail flick tests (**d,e**). (*P < 0.05, ^#^P < 0.01, ^¥^P < 0.001 compared with the MS group n = 6 in each group). OT was induced by repeated daily MS injections at 7.5 mg/kg in rats (**f,g**) or 10 mg/kg in mice (**h**). MSC-TP was administrated at Day 14 when OT had fully developed. Pain-like behavior was evaluated by von Frey filament in rats (**f**) and tail flick tests in rats (**g**) and mice (**h**). (*P < 0.05, ^#^P < 0.01, ^¥^P < 0.001 compared with the same day MS group; n = 6 in MS group; n = 9 in IT and IV groups). Data: mean ± s.e. IT: intrathecal; IV: intravenous. MS, morphine sulfate; PWT, paw withdrawal threshold.

Intrathecal or intravenous MSC-TP did not cause any behavioral change in normal rats (Supplementary Fig. 1e,f, P > 0.05, n = 6 for NS, n = 8 for MSC). In contrast, it significantly and consistently attenuated the development of OT. Both intrathecal and intravenous MSCs were remarkably effective (Fig. 2a–h). A one-time transplantation significantly mitigated OT for the whole course of the experiments of up to 26 days (Fig. 2b,c). The effects were almost identical when the transplantation was performed one day or 7 days before morphine treatment. These experiments were repeated separately by two groups of experimenters who were blinded to the treatments (Supplementary Fig. 2). The effects were further evaluated by the tail flick tests (Fig. 2d,e. Figure 2d, df = 3, F = 202.6; P < 0.0001, n = 6 for each group; Fig. 2e: df = 3, F = 131.7; P < 0.0001, n = 6 for each group). Consistent with the paw withdrawal experiments, MSCs significantly increased the MPE (%) regardless of the route and time of the transplantation, although to a lesser extent (Fig. 2d,e).

MSC-TP significantly reversed established OT when it was performed 14 days after daily morphine treatment (Fig. 2f–h. Figure 2f: df = 9, F = 40.13; P < 0.0001, n = 9 for IV or IT MSC; MS n = 6; Fig. 2g: df = 3, F = 139.3; P < 0.0001, n = 9 for IV or IT MSC; MS n = 6; Fig. 2h: df = 6, F = 14.95; P < 0.0001, MSC n = 8; MS n = 6). Morphine-induced OT reached its peak at day 12 of daily morphine injections in both the mechanical and thermal tests (Fig. 2f–h). Both intrathecal and intravenous MSCs significantly and consistently restored the sensitivity to morphine. This therapeutic effect took place rather rapidly and lasted for the whole course of the experiment to day 28 with no sign of waning. Within 2 days of transplantation, the PWT differences increased significantly in both transplantation groups, compared to the control group (P < 0.001). Similarly, intrathecal or intravenous MSCs significantly increased tail flick MPE from below 15% to above 30% on day 16 and above 50% on day 24 (Fig. 2g) (P < 0.05). The therapeutic effects were further tested in mice. Intravenous transplantation of MSCs significantly attenuated OT. Compared to the control group, the mean tail flick latencies were significantly increased in the transplantation group (Fig. 2h). Consistent with the rat experiments, this effect took place within 2 days of the transplantation.

**Figure 3:**
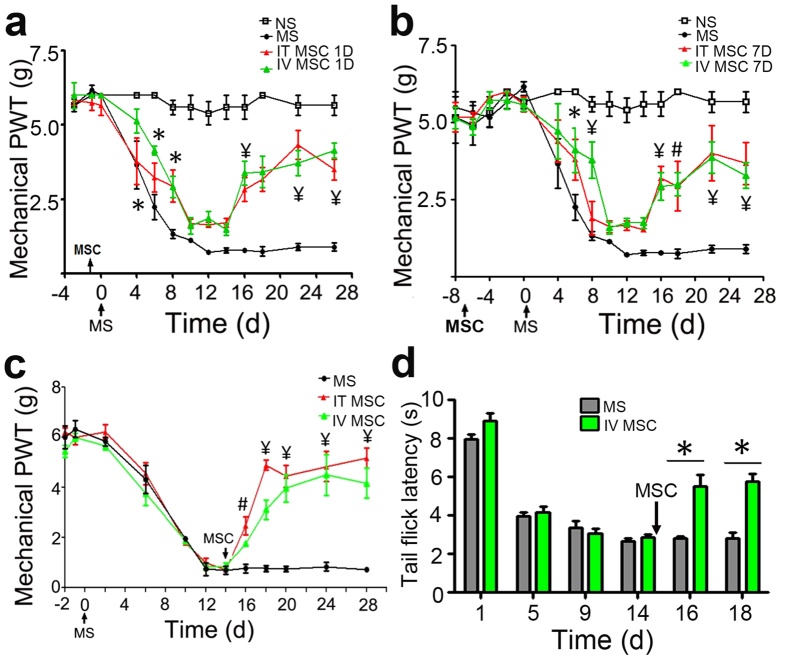
Preventive and therapeutic effects of MSC transplantation (MSC-TP) on opioid-induced hyperalgesia (OIH). MSC-TP was performed intrathecally (IT) or intravenously (IV) either 1 day (**a**) or 7 days (**b**) before the initiation of daily MS injections. MSC-TP of both routes significantly alleviated the morphine-induced reduction of PWTs, indicating preventive effects on OIH. (*P < 0.05, ^¥^P < 0.001 compared with the same day MS group; n = 6–8,). OIH was induced by daily MS injections of 7.5 mg/kg in rats (**c**) or 10 mg/kg in mice (**d**). MSC-TP was performed either intrathecally (IT) or intravenously (IV) at Day 14 in rats (**c**) or mice (**d**). Pain-like behavior was assessed by von Frey filament (**c**) or tail flick tests (**d**). MSC-TP of both routes consistently and significantly increased the mean PWT or the mean tail flick latency, indicating reversal of mechanical allodynia and thermal hyperalgesia in both rats and mice. *P < 0.05 compare to MS group (n = 5-8). Data: mean ± s.e. IT, intrathecal; IV, intravenous; MS, morphine sulfate; MSC-TP, MSC transplantation; NS: normal saline; PWT, paw withdrawal threshold.

Next, we tested the preventive and therapeutic effects of MSCs on OIH, which was also induced by daily morphine injections. OIH was reflected by the progressive decline of PWTs from the baseline values established 3 days before morphine treatment (Supplementary Fig. 1d, df = 17, F = 17.89; P < 0.0001. MS n = 1; NS n = 8). The decline took place over a course of 5–7 days (*P* < 0.001) and the hyperalgesia status persisted even after the cessation of daily morphine injections. Such decline was not seen in the control group, which received daily saline injections (*P* > 0.05). Intrathecal or intravenous MSC-TP (5 × 10^5^) substantially prevented the development of OIH (Fig. 3). The effects were long-lasting with no sign of waning over time. Consistent results were observed whether the transplantation was performed one day or seven days before morphine treatment. These results were replicated by two groups of experimenter who were blinded to the treatments. To test the therapeutic effects, MSC-TP was performed once OIH had fully developed. The transplantation effectively and rapidly reversed OIH. This effect lasted for the whole duration of the experiments (Fig. 3c, df = 11, F = 116.5; P < 0.0001, n = 6 for MS; n = 7 for IT or IV MSC). The therapeutic effect was further tested in mice (Fig. 3d, df = 5, F = 75.82; P < 0.0001. MS n = 6; MSC n = 8). Intravenous MSC-TP at day 14 of daily morphine treatment significantly increased the mean tail flick latency (P < 0.001).

**Figure 4:**
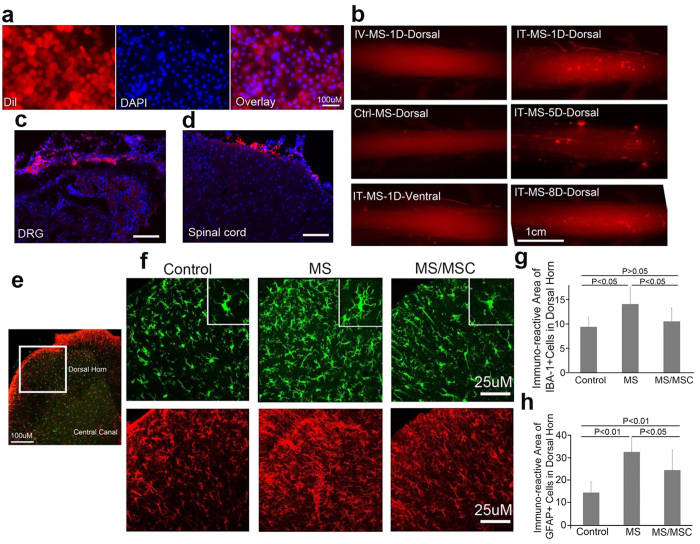
Tracing of transplanted MSCs and changes of microglia and astrocytes. (**a–d**) Fates of MSCs after intrathecal and intravenous transplantations. **(a)** Microscopy of labeled MSCs before injection. MSCs were labeled with Dil dye (red, left) and DAPI for nucleus (blue, middle). Double labeling of MSCs is shown on the right. (**b**) Microscopy pictures of the lumbar area of the spinal cord, demonstrating MSCs residing in the pia mater of the dorsal side of the spinal cord, as well as in the surrounding of the DRGs, 1 to 8 days after transplantation. Cross section of the DRG (**c**) and the spinal cord (**d**) showing MSCs residing in the pia mater of the spinal cord and DRG 1 day after MSC-TP. Scale bar: 50 um (**a**), 1 cm (**b**), 50 um (**c,d). (e–h)** MSC-TP attenuated activation of microglia and astrocytes. (**e**) Area of focus in the spinal cord dorsal horn in rats. Scale bar: 100 um. (**f**) Immunostaining of IBA-1(green) and GFAP (red) in the dorsal horn. Compared to controls, daily MS injections activated microglial cells (MS, top middle) and astrocyte (MS, bottom middle). MSC-TP attenuated this process (MS/MSC). Inserted boxes (top) show zoomed out individual microglia. Scale bar: 25 μm. Quantification results (mean ± s.e.) of immune-reactive areas of IBA-1 (**g**) and GFAP (**h**). n = 12 for each group. IT, intrathecal; IV, intravenous; MS: morphine sulfate; MSC-TP, MSC transplantation.

All animals survived the entire course of the experiments up to 68 days and had normal locomotion, food and fluid intake, body weight gain, and biochemical parameters for liver and kidney functions (Supplementary Fig. 3, P > 0.05. NS group: n = 6, MS group n = 11, MS/MSC group n = 12). Histopathology examination at necropsy did not reveal any abnormality in any major organs. Dil labeled MSCs in red were successfully traced to the surface of the spinal cord and the dorsal root ganglia (DRG) after intrathecal transplantation (Fig. 4a–d). Double staining of Dil and DAPI (nucleus) confirmed viable MSCs on the dorsal side of the spinal cord and the DRGs at various time intervals. We did not find any Dil labeled MSCs in any of these tissues after intravenous transplantation.

Expressions of IBA-1 in microglia (green) and GFAP in astrocytes (red) in the spinal cord dorsal horn underwent significant changes in response to daily morphine injections and MSC-TP (Fig. 4e–h). The IBA-1 immuno-reactivity increased and the morphology of IBA-1 positive cells changed from ramified shape to an amoeboid shape in response to morphine treatment, evaluated at day 22 (Fig. 4f,g, control vs MS: 9.4 ± 2.0 vs 13.8 ± 4.6, P < 0.05, n = 12 for each group). MSC-TP largely restored the morphology to its resting state and decreased the IBA-1 immuno-reactivity to a level that was not significantly different from the control (Fig. 4f,g, MS vs MS/MSC: 13.8 ± 4.6 vs 10.5 ± 2.7; P < 0.05; control vs MS/MSC: 9.4 ± 2.0 vs 10.5 ± 2.7, P > 0.05, n = 12 for each group). In addition, the GFAP immuno-reactivity was significantly increased after morphine treatment and was substantially reduced in the transplantation group (Fig. 4f,h, Control vs MS: 14.4 ± 4 vs 33.7 ± 9.9, P < 0.01; MS vs MS/MSC: 33.7 ± 9.9 vs 24.4 ± 9.1, P < 0.05; control vs MS/MSC: 14.4 ± 4 vs 24.4 ± 9.1, P < 0.01, n = 12 for each group).

## Discussion

The search for effective preventive and therapeutic strategies to counteract OT and OIH has been invigorated by the gravity of the profound negative impacts of OT and OIH. Here we for the first time report a powerful anti-tolerance effect of MSC-TP. Both intrathecal and intravenous MSC-TPs effectively attenuated the development of OT when performed before the initiation of chronic daily morphine injections in rats. MSC-TP almost completely reversed chronic OT when performed after OT had been established, regardless of the route of transplantation. These findings were consistent in both rats and mice. Thus, we have provided several lines of evidence that MSC-TP is a promising preventive and therapeutic therapy for OT with great potentials for clinical translation.

We chose to focus on chronic OT, rather than acute OT, because it resembles more closely to clinical practice. In addition to the traditional approach to assessing OT by demonstrating a hallmark rightward shift in the agonist dose-response curve after 3 to 5 days of daily morphine administration (Supplementary Fig. 1a), we introduced a new paradigm to investigate chronic OT, which was induced by daily morphine injections for up to four weeks (Supplementary Fig. 1b–d). Preemptive MSC-TP significantly and persistently attenuated the development of OT (Fig. 2a–e). The results from two groups of investigators were strikingly consistent. The first group performed intrathecal MSC-TP and found a significant anti-tolerance effect (Supplementary Fig. 2). The second group further performed both intrathecal and intravenous MSC-TPs and confirmed the anti-tolerance effects by both routes of transplantation (Fig. 2a–e). In addition, we tested MSC-TP at two time points (1 day and 7 days) before morphine treatment and observed identical anti-tolerance effects (Fig. 2b,c). These data clearly indicate that MSC-TP, either intrathecally or intravenously, could effectively prevent the development of OT. In addition to the preventive effect, MSC-TP effectively reversed established OT and restored sensitivity to morphine (Fig. 2a,f–h). This finding is important because it suggests that the therapeutic effect may be applicable to an increasingly large population of patients with ongoing OT due to chronic use of opioids for a variety of cancer and non-cancer pain conditions. It is well known that analgesic tolerance is commonly developed but tolerance to opioid adverse effects, such as respiratory depression and constipation, does not readily develop. This differential tolerance is one of the most common reasons patients suffer from detrimental consequences including overdose and death when dose escalation is required to overcome analgesic tolerance. Managing this population is a daunting challenge even to well-trained pain specialists. MSC-TP promises to emerge as an effective therapy for OT. Its clinical translation may have a profound impact on improving the safety and efficacy of opioid therapy and reducing opioid overdose and death.

A second important finding of this work is the remarkable preventive and therapeutic effects of MSC-TP on OIH. We found a remarkable anti-hyperalgesia effect of MSC-TP in rats and mice. Similarly, the two groups of investigators independently showed consistent results and came to the same conclusions. The first group tested intrathecal MSC-TP and demonstrated a significant attenuation of the development of OIH. The second group tested both intrathecal and intravenous MSC-TPs and confirmed the preventive and therapeutic effects on OIH (Fig. 3). OIH and OT are related but distinct biological phenomena and clinical entities^24^. There is convincing evidence to clinically differentiate the two entities^9^. Dose escalation is required to overcome OT but such a strategy only further exacerbates OIH. In contrast, MSC-TP attenuated both OT and OIH.

Interestingly, intravenous MSC-TP resulted in similar degrees of anti-tolerance and anti-hyperalgesia effects compared to intrathecal MSC-TP (Figs 2 and 3). The former is clinically advantageous compared to the latter. However, since MSCs transplanted by this route are largely trapped in the lungs^25^,^26^,^27^ and may be injured by activation of complements^28^, we expected a short, if any, duration of the therapeutic effects. Surprisingly, both routes of MSC-TP achieved long-lasting preventive and therapeutic effects. This finding is clinically important and mechanistically intriguing. It not only indicates a convenient route of clinical application but also suggests systemic mechanisms of action. It doesn’t seem to be necessary to place the cells in close proximity to the spinal cord (Fig. 4a–d). MSCs likely exert anti-tolerance and anti-hyperalgesia effects through their powerful paracrine function, regulating the sensitivity to noxious stimulation and opioid medications through modulation of immune and inflammatory processes in the peripheral and central nervous systems.

Microglial activation in the spinal cord plays a prominent role in the development of both OT and OIH^14^,^29^. Attenuating this process by MSCs is a plausible explanation for the observed effects. Indeed, microglial activation, induced by daily morphine injections, was significantly attenuated by MSC-TP (Fig. 4e–g). Also notable was the upregulation of GFAP expression after morphine treatment (Fig. 4e,f,h). MSC-TP partially and significantly restored GFAP expression. These data are consistent with the reports that chronic morphine injection activated spinal and cortical glia cells^30^,^31^. Morphine tolerance and hyperalgesia/allodynia have been associated with spinal microglial and astroglial activation^32^. Selective activation of an astrocyte JNK pathway after the stimulation of neuronal μ-opioid receptor (MOR) appears to mediate astrocyte-neuron signaling and contribute to OIH^33^. Inhibition of spinal glial activation by fluorocitrate, a nonselective metabolic inhibitor of astrocytes, partially reversed the development of morphine tolerance in rats^31^. These observations support the notion that both immune cells (microglia) and glial cells (astrocytes) are involved in the development of OT and OIH. MSC-TP may have achieved its therapeutic effects through acting on these cells. In addition, MSCs may modulate other cell types in the innate and adaptive arms of the immune system. For example, MSCs shifted the cytokine secretion profile of dendritic cells, naïve and effector T cells [T helper 1 (TH1) and 2 (TH2)], and natural killer cells to a more anti-inflammatory phenotype^34^. Undoubtedly, our current mechanistic understanding of the MSC therapy is in its infancy. It is important to appreciate its complexity and resist the temptation of attributing the therapeutic effects to a single molecular signaling pathway because MSCs may regulate immune cells, glial cells, and neurons by mechanisms that include both direct cell-to-cell contacts and release of a multitude of soluble factors.

Our data suggest that MSC-TP is safe and practical. All animals survived the whole experiments up to 68 days and maintained normal locomotion, food and fluid intake, body weight gain, and liver and renal function parameters (Supplementary Fig. 3). Histology at necropsy did not reveal any abnormality in any major part of the body. Thus, there was no evidence of toxicity even with long-term experiments up to 68 days. The intrathecally transplanted cells may have survived and maintained function *in vivo* for at least 34 days (Figs 2 and 3). This is consistent with our finding that viable MSCs were found in the pia mater of the spinal cord (Fig. 4). The long survival and long-lasting effects of MSCs are particularly important in clinical applications. Several factors may have contributed to this success. We used MSCs from the bone marrow in an early passage (passage #4) and the cells are essentially non-immunogenic^34^,^35^,^36^. A recent study used cells after 16 passages and failed to demonstrate any analgesic or anti-inflammatory effects^11^. Applications of human MSCs are being explored extensively^37^ via multiple clinical trials on spinal cord injury^38^,^39^, cardiovascular disease^40^,^41^, Parkinson’s disease^42^, and diabetes^43^. Consistent with our results of xenogeneic transplantation from rats to mice (Figs 2 and 3), immune rejection has not been a major concern because MSCs are immune-privileged due to their absent or low expression of major histocompatibility complex class II (MHC-II) and other co-stimulatory molecules^44^. Human MSCs are viable in tissues for months after systemic administration in sheep^45^. MSCs are known to have a strong immunosuppressive property and have been used successfully in autologous as well as allogeneic MSC-TP without pharmacological immunosuppression^46^. This unique capacity is being utilized in combating autoimmune diseases in clinical trials^47^. In addition, it is well accepted that MSCs have extremely low risk of tumorigenicity^48^; MSCs could actually inhibit tumor growth^49^. Clinical studies have convincingly demonstrated that direct injection of MSCs does not produce unwanted side effects and is well tolerated and safe^50^,^51^.

In summary, we report a powerful anti-tolerance effect and a remarkable anti-hyperalgesia effect of MSC-TP in rats and mice. These effects were consistently observed by two groups of investigators independently. Both the intrathecal and intravenous routes of transplantation were effective. Intrathecally transplanted cells homed in the pia mater of the spinal cord and the DRGs and appeared to have maintained long-term viability. The animals showed normal vital functions without any trace of toxicity. The inhibitory effects of MSCs on microglia and astrocytes appeared to be related to the anti-tolerance and anti-hyperalgesia functions. It may be tempting to uncover a specific molecular or cellular mechanism of MSC action. However, it is most likely that multiple mechanisms are involved. MSCs may regulate immune cells and neurons by mechanisms that include both direct cell contact and release of soluble factors such as interleukin 10 (IL-10), leukemia inhibitory factor (LIF), and transforming growth factor (TGF) through a paracrine mechanism^52^,^53^. Activation of opioid receptors by MSCs may also contribute^54^. Collectively, we have demonstrated for the first time that MSC-TP promises to be an innovative, safe, efficacious, and cost-effective therapy to prevent and treat OT and OIH. This emerging therapy has enormous potential to profoundly impact clinical practice. It may improve the efficacy of opioid therapy, reduce the risk of opioid overdose, and save lives.

## Methods

The research protocols were approved by the Cleveland Clinic Institutional Animal Care and Use Committee. We used both rats and mice in this investigation in order to determine the consistency of our findings in different species with allogeneic and xenogeneic transplantations of MSCs. The methods were carried out in accordance with the approved guideline.

### Animals

Adult male Sprague–Dawley rats weighing 200–250 g, 8–10 weeks old (Harlan, Indianapolis, IN, USA) were used in experiments on opioid tolerance and opioid-induced hyperalgesia. The animals were group housed (2/cage) on a 12 hour light/dark cycle with food and water available ad libitum. The animals were allowed to habituate to the housing facilities for at least 1 week before starting behavioral experiments.

C57BL/6J mice (8–10 weeks, The Jackson Laboratory, Bar Harbor, Maine. USA) were used and were group housed (4–5/cage) in standard cages in a colony room maintained on a 12-hour reversed light/dark cycle. All mice had continuous access to food and water throughout the study. Similarly, mice were handled and adapted to the testing environment for 1 week prior to initiation of the experiments. All behavioral testing procedures were conducted between 08:00 and 13:00 h. Animals used in this study were cared for in accordance with the guidelines of the Institutional Animal Care and Use Committee at Cleveland Clinic.

### Isolation and culture of MSCs from the bone marrow of rats

MSCs were isolated from the bone marrow as described^55^ with minor modifications. Rats (n = 6) sacrificed by CO2 asphyxiation according to Institutional Animal Care and Use Committee (IACUC) guidelines. The femurs and tibiae were removed from six-week-old male Sprague-Dawley rats, and washed three times with sterilized 1xPBS. The ends of the tibia and femur were cut by sharp Scissors. A 25-gauge needle was inserted into the bone marrow to flush out the tissue with a-MEM and filtered through a 100-μm filter mesh (BD Bioscience). The bone marrow (BM) cells were cultured in a-MEM with 16% fetal bovine serum (FBS), 1% L-glutamine, and antibiotic solution (100 u/ml penicillin-streptomycin) in culture flask and incubated at 37 °C with 5% CO2. The medium was replaced after 24 h and every 3–4 days thereafter. MSCs were passaged when they reached 90% confluency by 1 min treatment with 0.05% trypsin and 0.02% EDTA at 37 °C. MSCs were characterized by surface markers through flow cytometry and by differentiation into adipogenic and osteogenic cells. Cell differentiation was tested at passage 4 according to manufacturer’s protocol (Rat MSC differentiation kit, sc020, Fisher). MSCs used in all experiments were controlled within passage 5.

### Characterization of MSCs by flow cytometry

MSCs were expanded to passage four and were examined for surface marker expression using flow cytometry as previously described^56^,^57^ with modifications. Briefly, cultured MSCs were harvested, washed, and re-suspended in FACS buffer (1% FCS and 0.1% sodium azide in 1xHBSS). After blocking with CD16/CD32 Abs at 4 °C for 30 min, cells were stained for surface markers with directly conjugated Abs in FACS buffer at 4 °C for 30 min. Cells were washed twice and re-suspended in 200–400 μl of PBS for flow cytometry analysis as described before^57^. Abs used in our experiments were CD31PE-CY7 (Clone: 390, eBoscience), CD44HFITC (Clone: OX-49, BD), CD90.1 BV711 (Clone OX-7, BD), CD45APC-CY7 (Clone: OX-1, BD), CD11bPE (Clone: OX-42, BD), CD29BV450 (Clone: HA2/5BD). Flow cytometry analysis was performed with a LSRFortessa cytometer (BD Biosciences) and equipped with CellQuest software (BD Biosciences), and 50,000 events were acquired. Data were analyzed with FlowJo software (Tree Star).

### Characterization of MSCs by differentiation

#### Adipogenic differentiation

MSCs were incubated in the completed culture medium supplemented with Rat MSC differentiation kit (sc020, Fisher) for 2 weeks. The medium was changed twice per week. After 2 weeks of adipogenic differentiation, the cells were washed with PBS and fixed in 4% PFA (paraformaldehyde, Sigma) for 1 h at 4 °C, stained for 10–15 minutes at room temperature with a working solution for Oil Red O (Sigma) stain, and rinsed three times with distilled water.

#### Osteogenic differentiation

MSCs were cultured in the completed culture medium supplemented with Rat MSC differentiation kit (sc020, Fisher). After three weeks of osteogenic differentiation, cells were fixed in 4% PFA (paraformaldehyde, Sigma) for 1 h at 4 °C and stained with 40 mM Alizarin Red (pH 4.1, Sigma) for 10 minutes to visualize calcium deposition.

### Induction of OT and OIH

#### Acute opioid tolerance

Rats were injected with equal volume of normal saline (control) or 7.5 mg/kg morphine (experimental group) subcutaneously for 3 days. On the fourth day, rats of both groups received cumulative doses of morphine (0.0, 3.2, 5.6, 8.0, 10.0, and 18.0 mg/kg) as described^58^. Thermal plantar test was used to construct the agonist dose-response curves.

#### Chronic OT and OIH

Morphine was diluted in sterile saline solution (0.9% NaCl) to a concentration of 7.5 mg/ml. Control group received an equal volume of sterile saline. Morphine sulfate (7.5 mg/kg) was injected subcutaneously daily to induce opioid tolerance. Either saline or morphine was injected at 10:00–11:00 AM.

To evaluate OT, paw withdrawal thresholds (PWTs) (in grams) to mechanical stimulation were measured before and 50 min after each daily morphine injection. The differences between the two measurements reflect the level of tolerance to morphine. A large difference indicates no or low tolerance while a small difference indicates high tolerance. Similarly, thermal stimulation (Hargreave’s test) and tail flick test were further used to evaluate opioid tolerance. The response latencies (in seconds) were measured before the initiation of daily morphine injections to establish baseline control values and then measured in various time points after the initiation of daily morphine injections (Figs 2, 3 and 4). The measurements were done in the morning, 30 min after each morphine injection.

OIH was assessed by measuring PWTs to mechanical stimulation. Baseline values were first established 3 days before the initiation of daily morphine injections. PWT values were then measured every other day after the initiation of morphine injections. All measurements were taken in the morning before each morphine injection.

#### OT and OIH in mice

To induce OT, mice were injected with 10 mg/kg of morphine for 14 days as previously reported with modification^59^. Tail flick tests were performed before the initiation of daily morphine injections to establish baseline values. The response latencies (in seconds) were further measured after initiation of daily morphine injections. The measurements were taken in the morning before and 30 min after each morphine injection. The differences between the two measurements reflect the level of tolerance to morphine. A large difference indicates no or low tolerance while a small difference indicates high tolerance.

OIH was assessed by measuring tail flick test. Baseline values were first established 3 days before the initiation of daily morphine injections. Tail flick latencies were then measured at defined intervals after the initiation of morphine injections.

### Intrathecal and intravenous transplantation of MSCs

Intrathecal transplantation of MSCs was performed in the lumbar region, as described by Lu *et al*.^60^ with modification. The rat was shaved in lumbar region of the back under anesthesia (40 mg/kg Phenobarbital Sodium), and placed on a rolled pad so the back was arched. After skin disinfection, the L4-L5 lumbar interspace was identified by palpating spinous processes. A needle (27G) was slowly advanced through the skin over the L4-L5 interspace until it reaches the subarachnoid space. Upon confirmation of needle placement in the subarachnoid space by a tail flick, a single dose of cells in 10 ul PBS was injected over a period of one minute.

Intravenous transplantation of MSCs was performed through the tail vein in rats and a lateral retro-orbital approach in mice^61^. One dose of MSCs in 100 ul PBS was injected in each animal.

MSC-TP was performed before the initiation of daily morphine injections to evaluate the preventive effect or after the initiation of daily morphine injections to evaluate the therapeutic effect. For preventive effect, transplantation was performed one day or 7 days before the initiation of daily morphine injections. At each experimental paradigm, rats were divided into three groups, each receiving PBS (Control group), 0.5 million MSCs intrathecally (IT MSC group), or 0.5 million MSCs intravenously (IV MSC group). Each group had 13–14 animals. Behavioral tests were performed on the days as indicated (Fig. 3a).

For therapeutic effect, transplantation was performed 14 days after the initiation of daily morphine injections when opioid tolerance and opioid-induced hyperalgesia have been established. Similarly, rats were divided into three groups, each receiving PBS (Control group), 0.5 million MSCs intrathecally (IT MSC group), or 0.5 million MSCs intravenously (IV MSC group). Each group had 13–14 animals. Mechanical test and tail flick for thermal test were performed on the days as indicated (Fig. 4a).

### Behavioral tests

The sensitivity to noxious stimulation was determined by measuring the paw withdrawal thresholds (PWTs) in response to mechanical and thermal stimulation to the right hind paw or by measuring the tail flick latencies in response to a set temperature thermal stimulation to the tail. Animals were handled and habituated before behavioral testing to familiarize them with the environment and to minimize stress. All behavioral tests were performed in the Behavior Core Facility by experienced experimenters who were blinded to the treatment. The behavioral measurements began prior to morphine treatment to determine the baseline values and continued as indicated (Figs 3a and 4a).

#### Mechanical Sensitivity Testing

Animals were placed in individual 10 × 10 × 15 cm plastic boxes on an elevated metal mesh floor and allowed to acclimate for at least 30 min before test. Mechanical sensitivity was tested using von Frey sensory evaluator filaments (*Stoelting, Wood Dale, IL, USA*)^62^,^63^. Filaments were applied to the plantar surface of the right hind paw in ascending order of force (0.4–60 grams) until the filament bent and was held there for ~3 seconds or until a paw withdrawal response took place. Upon a paw withdrawal response, the filaments were applied in descending order, beginning with the next thinner filament until there was no withdrawal response. The threshold was the thinnest filament to evoke a paw withdrawal response. The procedure was repeated three times at 5 min intervals to avoid sensitization and the withdrawal thresholds were averaged and recorded (mean ± SEM).

#### Thermal Sensitivity testing (Tail Flick Test)

Sensitivity to thermal stimulation was evaluated by the hot water tail-flick test^64^ with modification. The temperature of a digital water bath was set up (52 °C) and confirmed with a glass thermometer. Animals were allowed to acclimate to the laboratory environment for 1 h before testing. After subcutaneously injection of morphine or saline, the animals were returned to their cage. For tail flick test, the animals were restrained with a restrainer *(Harvard Bioscience, Inc., Whitehall, PA*). Approximately 5 min before the test, animals were removed from their cage and allowed to crawl into the restrainer. The tail was marked with ink at one third from the distal tip and submerged in the hot water to the marked level. The cut-off time was limited to 10 s to avoid the tissue damage as measured on a digital laboratory timer. The flick of the tail was recorded as the tail-flick latency in seconds. The test was performed 30 min after morphine injection. Data were calculated as percent maximal possible effect (100% MPE), which was calculated by the following formula: 100% × [(treatment response time – basal response time)/(10 s –basal response time)]  =  100% MPE. The group data were expressed as mean MPE ± SEM. The entire behavioral testing was blinded with respect to the treatment groups.

#### Thermal plantar testing (Hargreaves test)

Rats were allowed to habituate in the environment for at least 60 min prior to the behavioral test^65^. Each rat was placed in a box (22 × 12 × 12 cm) containing a smooth glass floor (*Stoelting, Wood Dale, IL, USA*)^62^,^66^. The temperature of the glass was measured and maintained at 27 °C ± 0.5 °C. A heat source (*Stoelting, Wood Dale, IL, USA*) was focused on a portion of the hindpaw and a radiant thermal stimulus was delivered. The stimulus shut off automatically when the hindpaw moved or 20 seconds had passed to prevent tissue injury. The intensity of the radiant heat stimuli was adjusted to obtain either short or long baseline latencies. This allowed quantization of the treatment effect (lengthening of the latency, relative to the baseline values and control groups). In this study, latencies for Hargreaves stimuli at baseline ranged from 7 to 11 s. The experimenters were blinded to group assignments.

#### The safety measures

The safety measures included food and fluid intake, locomotion, body weight (g), and liver and renal function tests. Since the locomotor function and food and fluid intake were not noticeably different between different treatment groups in our preliminary experiments, we focused on measuring and recording body weight gain (expressed as mean ± SEM) over time among different experimental groups. In addition, we also measured the levels of blood glucose and alanine aminotransferase (*ALT*) to monitor the liver function and blood urea nitrogen (BUN) and creatinine to monitor the kidney function.

### Immunohistochemistry and quantitation of immunoreactivity staining

Histology and immunohistochemistry of microglia and astrocytes in the spinal cord were performed as we have described previously^61^,^67^. Briefly, animals were sacrificed by intracardiac perfusion with ice-cold PBS, followed by 4% PFA solution under deep anesthesia (Phenobarbital Sodium). The lumbar segment of the spinal cord was rapidly dissected and post-fixed in 4% PFA. It was washed with PBS and placed in cryoprotection buffer overnight at 4 °C until it sank. Afterwards, 30-um-thick coronal sections of the spinal cord were cut on a sliding microtome or cryostat machine (Leica Microsystems) and kept in cryostorage buffer at −20 °C.

For immunohistochemistry staining, the tissue slices were rinsed in PBST (1xPBS + 0.05% TritonX-100), blocked by incubation with 5% goat serum and 1% BSA at room temperature for 1 h, and then incubated overnight at 4 °C with primary Abs for microglia specific marker IBA-1 (rabbit anti-mouse/rat IBA-1, 1:5000, WAKO) and astrocyte specific marker GFAP (mouse-GFAP-Cy3, 1:5000, C9205, Sigma). The next day, tissues were incubated with Alexa 594-anti-mouse antibody and Alexa 488-anti-rabbit antibody (Invitrogen) for 1 hour. The immunofluorescence stained tissues were mounted with VECTASHIELD HardSet Anti-fade mounting medium with DAPI (H-1500, Vector lab). Omission of the primary antibody served as negative control. The stained sections were examined with a fluorescence microscope (Leica, Wetzlar, Germany), and images were captured with confocal microscopy (Leica, Germany).

#### Quantitation of immunoreactivity and cell counting

The quantification of percentage area occupied by immunoreactivity, the number of cells in predetermined area of the superficial dorsal horn (number per unit area), or percentage of immunoreactive cells among total cells were performed as previously described^61^,^67^. Briefly, 40x pictures of the superficial area of the lumbar spinal cord dorsal horn (L4-6) were captured by confocal microscopy and used for quantification. Digitized images were analyzed with National Institutes of Health ImageJ1.34s software. A thresholding procedure was established to determine the proportion of immunoreactive area within each fixed field of view. These parameters were then held constant for each set of images obtained at equal objectives and light intensities. The data represent the mean immunoreactivity area in the spinal cord horn. The percentage of total number of microglial cells was counted in defined area of the spinal cord dorsal horn (Fig. 11).

### MSCs labeling and tracing *in vivo*

MSCs were labeled with Dil (Invitrogen, D-3911) according to manufacturer’s instructions. In brief, 2 to 3 million of MSCs were collected and incubated with Dil dye (2 mg/ml) at 1XPBS w/o Ca^2+^ and Mg2^+^ buffer (Invitrogen) at 37 °C water bath for 5 minutes with 2–3 times of shaking. Cells were washed with the 1xPBS and re-suspended in 1xPBS for transplantation. Rats lumbar spinal cord segments, L1–L6 DRGs and sciatic nerve were perfused with 4% PFA indicated below after MSC-TP 1, 5 and 8 days. Low magnification pictures (10x) for the whole spinal cord were captured by anatomy fluorescence microscope with digital camera (Leica, Image Core, CCF, LRI). The tissues were cut into 20 uM section to exam Dil histologically.

### Statistical analysis

The paw withdrawal thresholds to mechanical stimulation were expressed as mean ± SEM. The withdrawal thresholds to thermal stimulation (Hargreaves test or tail flick test) were used to calculate % MPE as described and expressed as the mean % MPE ± SEM. The use of % MPE took into account the inevitable differences in baseline values between different groups of animals so that such variability would not affect comparisons between different groups. Body weight and blood tests for liver and kidney functions were expressed as mean ± SEM. Immunoreactivity was expressed as mean ± SEM.

Statistical analyses were made using one way or two way analysis of variance (ANOVA) followed by paired comparisons with Bonferroni corrections when comparisons were made between more than 3 groups. One way analysis of variance (ANOVA) followed by Turkey’s multiple comparison test for immunohistochemistry analysis. Graphpad Prism was used for all the analysis. P < 0.05 was considered statistically significant (*P < 0.05; ^#^P < 0.01; ^¥^P < 0.001).

## Additional Information

**How to cite this article**: Hua, Z. *et al*. Mesenchymal Stem Cells Reversed Morphine Tolerance and Opioid-induced Hyperalgesia. *Sci. Rep.* **6**, 32096; doi: 10.1038/srep32096 (2016).

## Supplementary Material

Supplementary Information
