# Corrigendum: Mesenchymal Stem Cells Reversed Morphine Tolerance and Opioid-induced Hyperalgesia

**DOI:** 10.1038/srep40978

**Published:** 2017-01-19

**Authors:** Zhen Hua, LiPing Liu, Jun Shen, Kathleen Cheng, Aijun Liu, Jing Yang, Lina Wang, Tingyu Qu, HongNa Yang, Yan Li, Haiyan Wu, John Narouze, Yan Yin, Jianguo Cheng

Scientific Reports 6: Article number: 32096; 10.1038/srep32096 published online: 08 24 2016; updated: 01 19 2017.

The original version of this Article contained a typographical error in the spelling of the author Kathleen Cheng, which was incorrectly given as Katherine Cheng. This has now been corrected in the PDF and HTML versions of the Article.

