# Supplementary material for: Mesenchymal Stem Cells Reversed Morphine Tolerance and Opioid-induced Hyperalgesia
Source: Sci Rep. 2016 Aug 24;6:32096. doi: 10.1038/srep32096 (PMC4995471; doi:10.1038/srep32096)
Supplement: Supplementary Information [file srep32096-s1.pdf]

Supplementary information to

Mesenchymal Stem Cells Reversed Morphine Tolerance and Opioid-induced Hyperalgesia

Zhen Hua[1,2] *, LiPing Liu[1]*, Jun Shen[1], Katherine Cheng[1], Aijun Liu[1], Jing Yang[1], Lina Wang[1], Tingyu Qu[3], HongNa Yang[3], Yan Li[3], Haiyan Wu[1], John Narouze[1], Yan Yin[1], Jianguo Cheng[1]

1. *Departments of Pain Management and Neurosciences, Lerner Research Institute and Anaesthesiology Institute, Cleveland Clinic, 9500 Euclid Avenue, Cleveland, Ohio, USA 44195*
2. Department of Anesthesiology, Beijing Hospital, No.1 Dahua Road, Beijing, China 100730
3. *Psychiatric Institute, Department of Psychiatry, College of Medicine, University of Illinois at Chicago, Chicago, IL, USA*

* Drs. Hua and Liu equally contribute to this work.

Correspondence:
Jianguo Cheng, MD, PhD
Professor of Anesthesiology and Director of Pain Medicine Fellowship Program
Departments of Pain Management of Neurosciences, Cleveland Clinic
9500 Euclid Avenue/C25
Cleveland, Ohio, 44195
chengj@ccf.org

Running head: Stem cells reversed morphine tolerance and hyperalgesia

Introductory paragraph word count: 195; Text word count: 1499; Figures: 4; Supplementary Figures: 3; Table: 0; References: 34

Supplement Figure 1

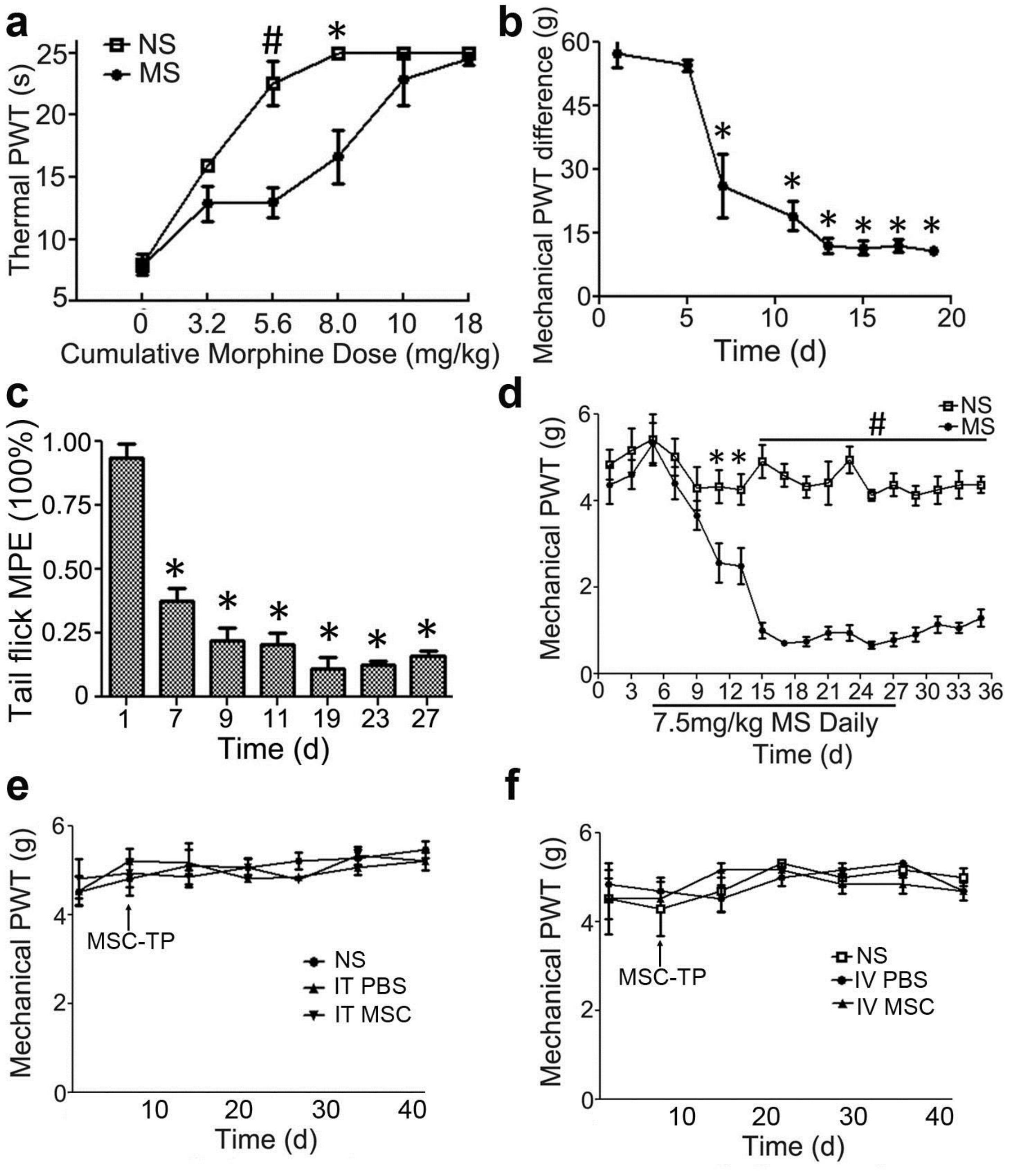

Supplement Figure 1. **Induction of opioid tolerance (OT) and opioid-induced hyperalgesia (OIH). (a)** Acute tolerance. Agonist dose-response curves were constructed with increasing doses of morphine (MS) (0–18 mg/kg) in rats that had received 3 days of daily injections of normal saline (NS) or MS (7.5mg/kg). The Plantar test was used to construct the dose-response curves 30 min after third day of daily NS or MS injection. (MS: n=5 and NS: n=3, thermal

response cutoff time: 25s; #P<0.01; *P<0.05 between groups). **(b)** Chronic tolerance assessed by mechanical stimulation. Rats were treated with MS daily for 3-4 weeks. Paw withdrawal thresholds to mechanical stimulation by von Frey filaments were tested before and 50 min after MS injection. The difference between the two measurements indicates responsiveness to MS. A large difference indicates low or no tolerance while a small difference indicates high tolerance (n=8, *P<0.05 compared the mean value recorded in Day 1 of MS injection). (**c**) Chronic tolerance assessed by tail flick test. Maximum possible effect (MPE) of MS was used to indicate tolerance. The lower the MPE (%) the higher the tolerance (n=12, *P < 0.05 compared the mean value recorded in Day 1 of MS injection). (**d**) Chronic OIH. PWTs were assessed by von Frey filament before daily MS injections. The progressive declining of PWTs indicates hyperalgesia in response to daily morphine injections (n=6, #P<0.01; *P<0.05 compare to the NS control group). (**e, f**) In normal rats, PWTs did not change in response to intrathecal (**e**) or intravenous (**f**) injection of PBS or MSCs (0.5x 10^6) (n=6-8). Data: mean± s.e. IT, intrathecal; IV, intravenous; MPE, maximal possible effect; MS, morphine sulfate; NS, normal saline; PWT, paw withdrawal threshold.

Supplement Figure 2

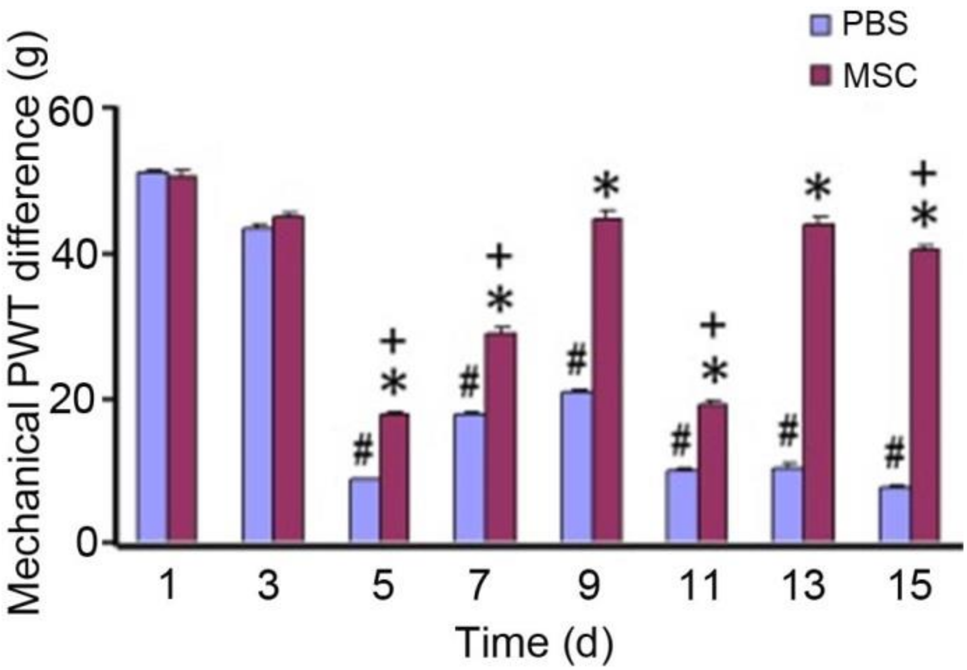

Supplement Figure 2. MSC-TP reversed OT induced by daily morphine injections in rats. Decreasing PWT difference between the measurements made before and 30 min after MS injection indicates OT. * P<0.05 compared with same day PBS; # P<0.05 compared with PBS Day 1; + P<0.05 compared with MSC Day 1. n=12 in each group. Please note the sham control group (injection with saline) was not included for clarity. The effect of morphine injection is reflected by increased PWTs (larger Y axis scale).

Supplement Figure 3

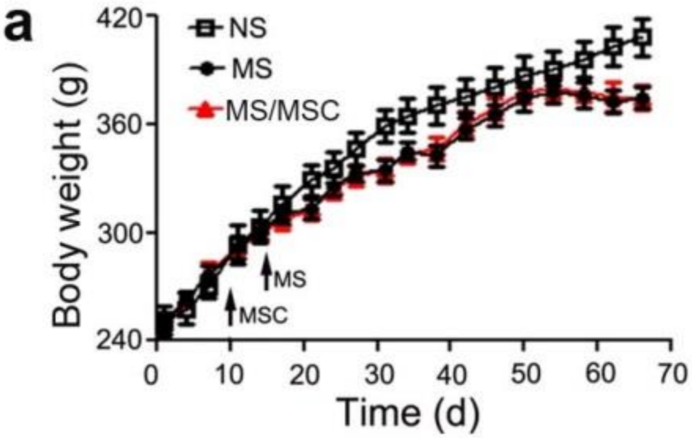

b

Group	ALT(U/L)	Cholesterol (mg/dL)	Glucose(mg/dL)	BUN(mg/dL)	Creatinine(mg/dL)
NS	63.7±3	92.7±3	155.0±9	18.0±1.1	0.2±0
MS	70.3±3	91.3±7	151.7±10	18.6±2.2	0.2±0
IT MSC	60.0±9	86.3±3	143.3±10	18.4±2.5	0.3±0.1
IV MSC	75.8±6	96.3±5	152.8±21	18.6±2.8	0.2±0.2

Supplement Figure 3. Long term safety of MSC transplantation (MSC-TP). (**a**) Body weight gain was not affected by MSC-TP. Daily MS injections slightly but significantly reduced body weight gain compared to the NS control group (P<0.05 in many time-points between Days 24 and 68). However, there were no significant differences between the MS group and the MS+MSC group (P>0.05). NS group n=6, MS group n=11, MS+MSC group n=12. Data mean± s.e. (**b**) Normal liver and kidney functions after long-term MSC-TP. Blood plasma was collected from rats at the end of the experiments (55 days after MSC-TP). Biochemical tests for liver function (ALT, cholesterol and glucose) and kidney function (BUN, creatinine) were performed. Data: mean± s.e. P>0.05. n=6 each group. MS, morphine sulfate; MSC, mesenchymal stem cell; NS: normal saline.
